# Classical blood biomarkers identify patients with higher risk for relapse 6 months after alcohol withdrawal treatment

**DOI:** 10.1007/s00406-020-01153-8

**Published:** 2020-07-05

**Authors:** Florian J. Raabe, Elias Wagner, Judith Weiser, Sarah Brechtel, David Popovic, Kristina Adorjan, Oliver Pogarell, Eva Hoch, Gabriele Koller

**Affiliations:** 1 Department of Psychiatry and Psychotherapy, University Hospital, LMU Munich, Nussbaumstrasse 7, 80336 Munich, Germany; 2 International Max Planck Research School for Translational Psychiatry (IMPRS-TP), Kraepelinstrasse 2-10, 80804 Munich, Germany; 3 Institute of Psychiatric Phenomics and Genomics (IPPG), University Hospital, LMU Munich, Nussbaumstrasse 7, 80336 Munich, Germany; 4 Division of Clinical Psychology and Psychological Treatment, Department of Psychology, LMU Munich, Leopoldstrasse 13, 80802 Munich, Germany

**Keywords:** Alcohol dependence, Alcohol use disorder, Withdrawal treatment, Risk for relapse, Outcome, Blood biomarkers

## Abstract

This naturalistic study among patients with alcohol dependence examined whether routine blood biomarkers could help to identify patients with high risk for relapse after withdrawal treatment. In a longitudinal study with 6-month follow-up among 133 patients with alcohol dependence who received inpatient alcohol withdrawal treatment, we investigated the usefulness of routine blood biomarkers and clinical and sociodemographic factors for potential outcome prediction and risk stratification. Baseline routine blood biomarkers (gamma-glutamyl transferase [GGT], alanine aminotransferase [ALT/GPT], aspartate aminotransferase [AST/GOT], mean cell volume of erythrocytes [MCV]), and clinical and sociodemographic characteristics were recorded at admission. Standardized 6 months’ follow-up assessed outcome variables continuous abstinence, days of continuous abstinence, daily alcohol consumption and current abstinence. The combined threshold criterion of an AST:ALT ratio > 1.00 and MCV > 90.0 fl helped to identify high-risk patients. They had lower abstinence rates (*P* = 0.001), higher rates of daily alcohol consumption (*P* < 0.001) and shorter periods of continuous abstinence (*P* = 0.027) compared with low-risk patients who did not meet the threshold criterion. Regression analysis confirmed our hypothesis that the combination criterion is an individual baseline variable that significantly predicted parts of the respective outcome variances. Routinely assessed indirect alcohol biomarkers help to identify patients with high risk for relapse after alcohol withdrawal treatment. Clinical decision algorithms to identify patients with high risk for relapse after alcohol withdrawal treatment could include classical blood biomarkers in addition to clinical and sociodemographic items.

## Introduction

Alcohol use disorder (AUD) is a severe, chronic substance use disorder with a significant individual and socio-economic burden; it is highly prevalent and affects approx. 100 million people globally [12]. Furthermore, about 40–60% of patients with AUD are presumed to relapse within the first year after treatment [11, 27]. Due to limited resources of health care systems, the effective allocation of medical treatment to individual patients is a major challenge [36]. A promising strategy to improve the outcome might be to individualize treatment and to identify patients on the basis of applicable subgroups and endophenotypes [24].

Biological and non-biological variables (e.g. sociodemographic, environmental and clinical factors) have been previously associated with the risk of relapse in patients or subgroups of patients with AUD [1, 8, 13, 29, 32, 38]. Despite neuroimaging [8, 13], genetics [29] and the measurement of neurometabolites [29] being sophisticated methods to estimate resilience and the risk of relapse after treatment, routine blood biomarkers might be a more feasible and cost-effective means of assessing relapse risk. The classical indirect alcohol blood biomarkers are the liver enzymes γ-glutamyl transferase (GGT), alanine aminotransferase (ALT), aspartate aminotransferase (AST) and the mean cell volume of erythrocytes (MCV). All of them reflect chronic excessive drinking and are basic parameters for clinical laboratories in daily routine care settings [3, 25]. A previous study investigated the ability of a combination of an AST:ALT ratio > 1.00 and an MCV > 90.0 fl to identify patients with alcohol dependence (AD) in a cohort of individuals with and without physical diseases and with and without elevated transaminase levels [17]. The combination of these routine blood biomarkers resulted in a remarkably high sensitivity of 97.3% and specificity of 88.9% [17].

So far, most studies have investigated the use of blood biomarkers (1) to identify patients with AD and/or (2) as a screening tool for potential alcohol consumption before sample collection and/or (3) to detect relapse and monitor abstinence in patients with AD [2, 3, 20, 42].

Blood biomarkers might not only help to identify patients with AD or help to monitor therapeutic strategies and abstinence but might also serve as prognostic markers to assess the risk of relapse and thus might support future clinical decision algorithms [31]. Indirect alcohol biomarkers represent an objective measurement for chronic cellular impairment that correlates with previous alcohol consumption [35] and, therefore, might reflect the severity of AD and consequently the risk of relapse after withdrawal treatment. In this context, in a sample of individuals with alcohol abuse and dependence, higher levels of the blood biomarker CDT and the liver function enzymes ALT and GGT were predictors for drunk-driving recidivism [23]. Furthermore, higher GGT scores were associated with unfavourable outcomes during and after pharmacotherapy for AD [15, 16]. In a longitudinal study assessing post-treatment outcomes in an outpatient sample with one-year follow-up after treatment, higher GGT, AST, ALT, MCV scores and AST/ALT ratios were associated with unfavourable outcomes with regard to drinking-behaviour [14].

In this study, we investigated the potential of biological (i.e. routine blood biomarkers, breath alcohol and alcoholic liver disease) and non-biological factors (i.e. sociodemographic and clinical variables) to estimate the risk for relapse after inpatient withdrawal treatment. Therefore, we conducted a naturalistic longitudinal study among patients with AD in a routine care setting with a 6-month follow-up to evaluate whether baseline both biological and non-biological factors, assessed at admission to inpatient alcohol withdrawal treatment, are associated with long-term outcome after completion of treatment.

## Methods

### Design

Included patients received routine inpatient care with a standardized multimodal and multi-professional qualified (extended) withdrawal treatment (QWT) at the inpatient addiction unit at the Department of Psychiatry and Psychotherapy, University Hospital, LMU Munich, Munich, Germany. Information on biological and non-biological baseline variables at admission was obtained from standardized electronic medical records. Previous inpatients were contacted and interviewed 6 months after completion of treatment to assess relevant outcome variables. We chose 6 months as the follow-up period since this is presumed to be a good predictor for 5-year outcome [40]. The study was approved by the local ethics committee of the Faculty of Medicine, LMU Munich, Germany (project number 585-16).

### Participants

The study was conducted between September 2016 and January 2018. Inclusion criteria were: (1) patients with a confirmed diagnosis of AD (F10.2) according to the *International Classification of Diseases and Related Health Problems 10th Revision* (ICD-10), (2) treated at the inpatient addiction unit between March 18, 2016 and July 26, 2017, (3) age ≥ 18 years and (4) participation in QWT for ≥ 5 days.

**Fig. 1:**
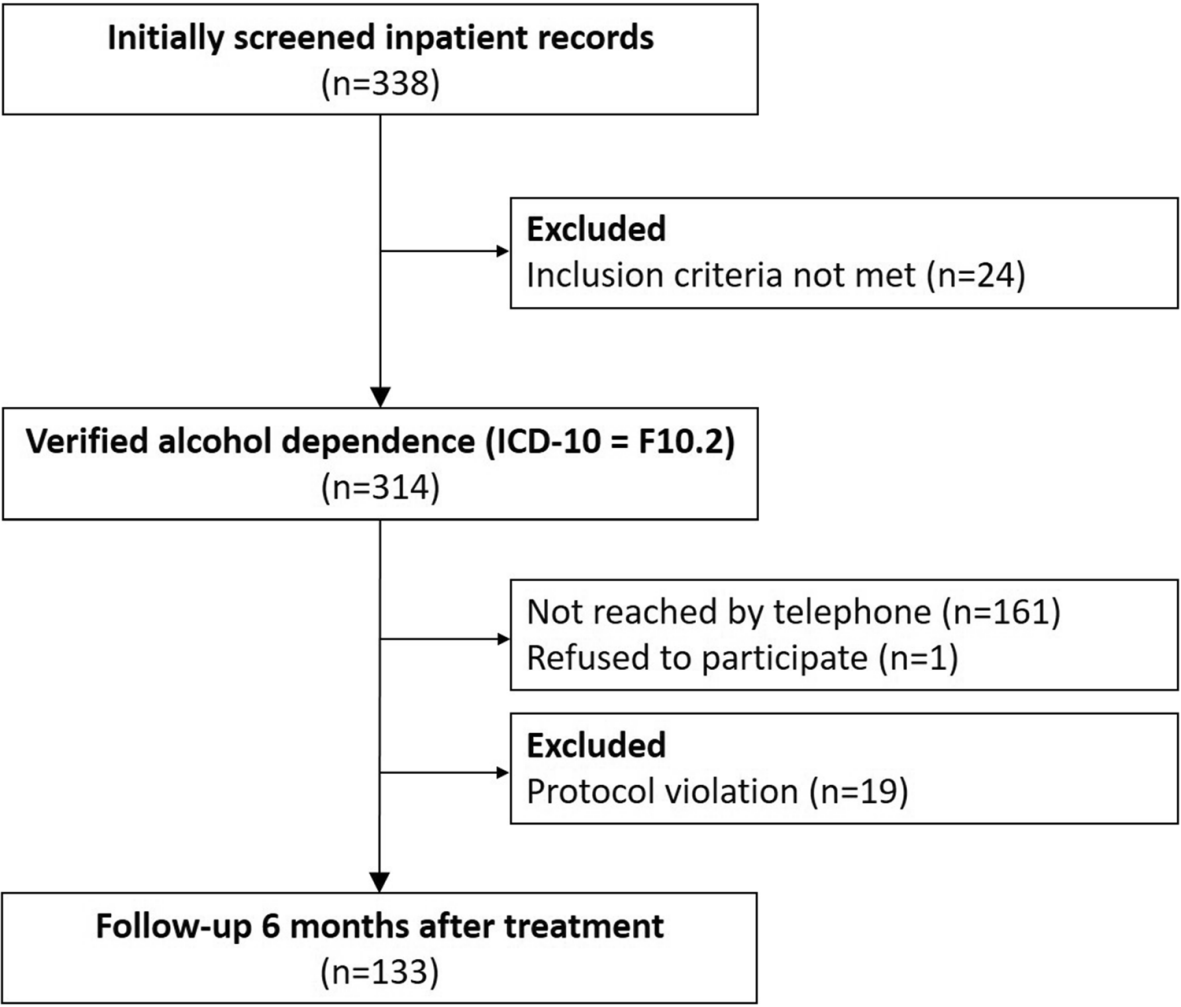
Overview of the selection process for the longitudinal study with 6-month follow-up among 133 patients with alcohol dependence who received inpatient alcohol withdrawal treatment

On the basis of electronic records, we identified 338 patients (71.3% male, 28.7% female, mean [SD] age: 46.7 [12.6] years) with a recorded diagnosis of AD according to ICD-10 who had received ≥ 5 days of QWT in the period of interest. To exclude patients with a diagnosis other than AD (e.g. multiple drug abuse [F19.2]), trained physicians re-assessed each patient’s diagnosis and excluded 24 patients with a diagnosis other than AD (e.g. multiple drug abuse [F19.2]). Thus, we contacted 314 former inpatients. 51.2% (161 out of 314) of previous inpatients could not be reached and 1 person (0.3%) declined to participate in the follow-up interview. Of the successfully contacted people, 19 were excluded due to a protocol violation (they were contacted earlier than 6 months after QWT). Thus, a total of 133 out of 314 (42.4%) former inpatients (71.4% males, 28.6% females, mean [SD] age 47.4 [13.0] years) were contacted successfully 6 months after QWT and interviewed by telephone. Figure 1 shows a detailed overview of the selection process.

Only two baseline features significantly differed between the study participants who were successfully contacted after 6 months and those who were not reached by telephone or were excluded (non-follow-up sample). More former inpatients in the non-follow-up group had an immigration background than in the participant group (43.1% vs. 26.1%, *χ*^2^ = 4.263, *df* = 1, *P* = 0.039) and the prevalence of alcoholic liver disease detected by ultrasound was higher in the non-follow-up group compared to the participant group (63.1% vs. 49.2%, *χ*^2^ = 5.682, *df* = 1, *P* = 0.017). All other baseline features including liver enzymes (AST, ALT, GGT) and MCV did not significantly differ between the follow-up group and the non-follow-up group.

## Setting

QWT is a multimodal and multi-professional inpatient detoxification and treatment programme [5]. It is usually planned for 14–21 days and is a highly standardized part of routine clinical care in the inpatient addiction unit at the Department of Psychiatry and Psychotherapy, University Hospital, LMU Munich, Munich, Germany. Medical treatment to control acute alcohol withdrawal symptoms included the application of benzodiazepines (oxazepam) and alpha-2 noradrenergic agonists (clonidine). Patients received medication as needed, according to their clinical condition. Patients with a history of at least one epileptic seizure were additionally treated with a fixed dose of anticonvulsants (carbamazepine or levetiracetam). Patients also received their previously prescribed medication for their physical and psychiatric conditions. In addition to symptom-specific or prophylactic treatment of withdrawal symptoms, QWT includes psychotherapeutic interventions covering motivational enhancement, cognitive behavioural therapy and relapse prevention; occupational therapy; physiotherapy; work-related therapy, art and music therapy; and attendance at self-help groups (e.g. AA groups). Abstinence during QWT was monitored with breath analysers for alcohol.

### Baseline data

Baseline data at admission to inpatient treatment were extracted from standardized electronic medical records. At admission, all patients were interviewed with a standard questionnaire based on the European Addiction Severity Index (EuropASI) [19], a semi-structured assessment instrument for drug and alcohol abuse. The clinical records of study participants during QWT were used to assess baseline data of the patients. We collected baseline data on both biological (routine blood biomarkers [GGT, AST, ALT, MCV], breath alcohol and alcoholic liver disease detected by ultrasound) and non-biological factors (sociodemographic and clinical variables; see Table 1 for details). We used the combined threshold criterion of an AST:ALT > 1.00 and a MCV > 90.0 fl [17] to divide participants into “low-risk patients”, who did not fulfil the criterion, and “high-risk patients”, who did. Baseline blood samples were collected within 72 h after admission and an ultrasound scan was performed to detect alcoholic liver diseases. Whole blood anticoagulated with EDTA was used to assess MCV with an automated haematology analyser and serum was used to determine y-GT, ALT and AST activity. All blood measurements were conducted at the Institute of Laboratory Medicine, University Hospital Munich.

**Table 1:** Associations of baseline factors with outcome variables 6 months after withdrawal treatment

Baseline factor *n* or *x̄* (SD) *n*_total_ = 133 unless otherwise indicated		Outcome variable									
		Current abstinence*, *n* or *x̄* (SD**)**		Statistics	Continuous abstinence*, *n* or *x̄* (SD)		Statistics	Daily alcohol consumption in g/d, *x̄* (SD) or *ρ*	Statistics	Days of continuous abstinence *x̄* (SD) or *ρ*	Statistics
Whole cohort		Yes 103	No 30		Yes 59	No 74		20.47 (57.40)		111.44 (72.17)	
Sociodemographic variables											
Sex Male Female	95 38	74 29	21 9	*χ*^2^ = 0.039, *df* = 1 *P* = 0.844	45 14	50 24	*χ*^2^ = 1.219, df = 1 *P* = 0.270	25.05 (66.33) 9.00 (19.67)	*U* = 1792.5 *P* = 0.932	115.21 (71.79) 102.00 (73.22)	*U* = 1605.0 *P* = 0.297
Age, years	47.40 (12.99)	46.90 (13.28)	49.10 (12.03)	*U* = 1411.0 *P* = 0.470	48.27 (13.75)	46.70 (12.41)	*U* = 2001.5 *P* = 0.411	*ρ* = 0.041	*P* = 0.642	ρ = 0.081	*P* = 0.355
Education Secondary school to end of year 9 Secondary school to end of year 10 Secondary school to end of year 12/13 University/college	(*n* = 121) 33 29 19 40	22 25 11 **36**	11 4 8 **4**	***χ***_**e**_^**2**^** = 10.997** ***P***_***FWE***_** = 0.010**	12 13 7 22	21 16 12 18	*χ*^2^ = 3.120, *df* = 3 *P*_*FWE*_ = 0.373	33.74 (79.05) 8.22 (25.25) 44.44 (88.23) **6.63 (25.06)**	***H = 11.113*** ***df = 3*** ***P***_***FWE***_** = 0.011**	104.12 (74.10) 112.97 (73.58) 113.84 (64.31) 117.08 (73.71)	*H* = 1.416 *df* = 3 *P*_*FWE*_ = 0.702
Living situation With partner/family/children Alone Flat-sharing community Assisted living Emergency shelter	(*n* = 130) 53 63 2 4 8	41 50 2 4 4	12 13 0 0 4	*χ*_e_^2^ = 4.222 *P*_*FWE*_ = 0.312	24 28 2 2 3	29 35 0 2 5	*χ*_e_^2^ = 2.438 *P*_*FWE*_ = 0.722	20.88 (60.51) 15.07 (44.40) 0 (0) 0 (0) 78.13 (113.49)	*H* = 6.426 *df* = 4 *P*_*FWE*_ = 0.169	113.89 (71.11) 111.86 (73.75) 180 (0) 123.00 (81.56) 94.00 (75.51)	*H* = 2.397 *df* = 4 *P*_*FWE*_ = 0.663
Partnership No Yes	(*n* = 131) 75 56	59 43	16 13	*χ*^2^ = 0.066, *df* = 1 *P* = 0.798	32 26	43 30	*χ*^2^ = 0.184, *df* = 1 *P* = 0.668	21.71 (63.09) 18.80 (50.32)	*U* = 2061.0 *P* = 0.803	111.32 (72.42) 112.11 (72.01)	*U* = 2047.0 *P* = 0.796
Immigration background No Yes	(n = 88) 65 23	51 19	14 4	*χ*_e_^2^ = 0.180 *P* = 0.672	25 9	40 14	*χ*^2^ = 0.003, *df* = 1 *P* = 0.955	19.10 (60.68) 28.13 (72.41)	*U* = 728.0 *P* = 0.793	104.69 (73.34) 101.83 (75.55)	*U* = 739.5 *P* = 0.938
Employment No Yes	(*n* = 130) 69 61	50 51	19 10	*χ*^2^ = 2.319, *df* = 1 *P* = 0.128	26 32	43 29	*χ*^2^ = 2.861, *df* = 1 *P* = 0.091	31.04 (74.30) 8.85 (26.55)	*U* = 1848.5 *P* = 0.101	**102.41 (75.90)** **122.97 (66.49)**	***U = 1676.5*** ***P = 0.036***
Clinical variables											
Duration of hospitalization	16.47 (6.42)	16.23 (6.30)	17.30 (6.86)	*U* = 1384.5 *P* = 0.382	17.05 (7.05)	16.01 (5.88)	*U* = 2385.5 *P* = 0.354	*ρ* = 0.069	*P* = 0.427	*ρ* = 0.073	*P* = 0.403
Type of discharge Regular Disciplinary Against medical advice	105 3 25	82 2 19	23 1 6	*χ*^2^ = 0.255 *df* = 2 *P*_*FWE*_ = 0.880	51 1 7	54 2 18	*χ*^2^ = 3.613 *df* = 2 *P*_*FWE*_ = 0.164	14.64 (42.79) 18.67 (32.33) 45.12 (96.54)	*H* = 0.405 *df* = 1 *P*_*FWE*_ = 0.525	118.28 (71.41) 74.00 (93.95) 87.2 (69.46)	*H* = 3.302 *df* = 1 *P*_*FWE*_ = 0.069
Mean (SD) dose of oxazepam needed between day 2 and 5, mg	72.61 (96.86)	**63.50** **(84.53)**	**103.92 (127.51)**	***U = 1023.5*** ***P = 0.041***	68.73 (83.89)	75.71 (06.54)	*U* = 2064.5 *P* = 0.579	***ρ = 0.191***	***P = 0.028***	*ρ* = 0.044	*P* = 0.619
Mean (SD) number of previous inpatient treatments	(*n* = 132) (4.77 [6.95])	4.78 (7.01)	4.76 (6.84)	*U* = 1470.5 *P* = 0.897	3.25 (3.92)	5.93 (8.51)	*U* = 1750.5 *P* = 0.058	*ρ* = 0.010	*P* = 0.909	***ρ = − 0.226***	***P =0 .009***
Psychiatric comorbidity (ICD-10 code; also see legend) None Depression (F32, F33) Anxiety disorder (F41) Personality disorder (F60) Other	59 46 3 12 13	44 37 2 9 11	15 9 1 3 2	*χ*_e_^2^ = 1.508 *P*_*FWE*_ = 0.841	27 22 0 3 7	32 24 3 9 6	*χ*_e_^2^ = 4.517 *P*_*FWE*_ = 0.339	23.54 (58.07) 12.61 (39.87) 101.33 (175.51) 27.90 (82.72) 8.76 (21.39)	*H* = 1.648 *df* = 4 *P*_*FWE*_ = 0.800	115.02 (69.01) 120.28 (70.47) 7 (7) 91.83 (73.98) 106.08 (84.42)	*H* = 8.828 *df* = 4 ***P***_***FWE***_ = 0.066
Previous alcohol delirium No Yes	122 11	96 7	26 4	*χ*_e_^2^ = 1.309 *P* = 0.267	54 5	68 6	*χ*_e_^2^ = 0.006 p = 1.000	19.39 (54.42) 32.40 (86.51)	*U* = 577.5 *P* = 0.297	109.16 (72.79) 136.64 (62.28)	*U* = 559.5 *P* = 0.340
Previous alcohol-related seizure No Yes	104 29	84 19	20 10	*χ*^2^ = 3.020, *df* = 1 *P* = 0.082	50 9	54 20	*χ*^2^ = 2.668, *df* = 1 *P* = 0.102	16.51 (52.97) 34.64 (70.32)	*U* = 1262.5 *P* = 0.068	113.09 (72.74) 105.52 (71.01)	*U* = 1362.5 *P* = 0.406
Tobacco use No Yes	50 83	39 64	11 19	*χ*^2^ = 0.014, *df* = 1 *P* = 0.905	26 33	24 50	*χ*^2^ = 1.894, df = 1 *P* = 0.169	12.87 (31.99) 25.04 (68.09)	*U* = 2036.5 *P* = 0.807	123.42 (73.37) 104.22 (71.52)	*U* = 1800.5 *P* = 0.182
Previous (il)legal drug abuse No Yes	90 43	68 35	22 8	*χ*^2^ = 0.568, *df* = 1 *P* = 0.451	42 17	48 26	*χ*^2^ = 0.600, *df* = 1 *P* = 0.439	23.78 (64.53) 17.37 (49.73)	*U* = 1814.0 *P* = 0.426	111.60 (73.13) 110.22 (71.78)	*U* = 1757.0 *P* = 0.370
Alcohol consumption before treatment, g/d	(*n* = 132) 191.76 (108.40)	188.28 (107.35)	204.15 (113.12)	*U* = 1361.0 *P* = 0.466	194.04 (120.70)	189.87 (97.82)	*U* = 2096.0 *P* = 0.792	*ρ* = 0.078	*P* = 0.377	*ρ* = − 0.016	*P* = 0.860
Biological variables											
Breath alcohol, **‰**	0.81 (0.80)	**0.74 (0.79)**	**1.05** **(0.80)**	***U = 1181.0*** ***P = 0.046***	0.70 (0.78)	0.90 (0.81)	*U* = 1812.5 *P* = 0.088	***ρ = 0.199***	***P = 0.022***	*ρ* = − 0.159	*P* = 0.068
Alcoholic liver disease No Yes	(*n* = 132) 67 65	51 51	16 14	*χ*^2^ = 0.103, *df* = 1 *P* = 0.748	29 29	38 36	*χ*^2^ = 0.024, *df* = 1 *P* = 0.878	23.78 (64.53) 17.37 (49.73)	*U* = 2115.5 *P* = 0.701	111.60 (73.13) 110.22 (71.78)	*U* = 2158.5 *P* = 0.928
yGT, U/l	271.59 (459.34)	251.46 (428.53)	340.70 (555.10)	*t*(131) = 0.936 *P* = 0.351	243.71 (387.02)	293.81 (511.27)	*t*(131) = 0.623 *P* = 0.534	*ρ* = 0.092	*P* = 0.292	*ρ* = − 0.008	*P* = 0.928
AST, U/l	87.37 (147.01)	87.70 (159.77)	86.23 (92.62)	*t*(131) = − 0.048 *P* = 0.962	84.00 (162.83)	90.05 (134.15)	*t*(131) = 0.235 *P* = 0.814	*ρ* = 0.075	*P* = 0.392	*ρ* = − 0.062	*P* = 0.477
ALT, U/l	71.11 (99.03)	73.32 (105.84)	63.50 (71.82)	*t*(131) = − 0.477 *P* = 0.634	72.15 (117.32)	70.27 (82.43)	*t*(131) = − 0.108 *P* = 0.914	*ρ* = − 0.024	*P* = 0.783	*ρ* = − 0.032	*P* = 0.718
AST:ALT ratio	1.23 (0.52)	1.18 (0.54)	1.37 (0.44)	*t*(131) = 1.724 *P* = 0.087	1.17 (0.46)	1.27 (0.56)	*t*(131) = 1.048 *P* = 0.296	***ρ = 0.232***	P = 0.007*	*ρ* = − 0.076	*P* = 0.383
MCV, fl	92.27 (5.38)	91.96 (5.47)	93.31 (5.01)	*t*(131) = 1.811 *P* = 0.228	91.31 (4.95)	93.03 (5.63)	*t*(131) = 1.844 *P* = 0.067	*ρ* = 0.106	*P* = 0.223	*ρ* = − 0.153	*P* = 0.079
AST:ALT > 1.00 + MCV > 90.0 fl criterion Not fulfilled Fulfilled	74 59	**65** **38**	**9** **21**	***χ***^**2**^** = 10.317, df = 1** ***P = 0.001***	38 21	36 38	*χ*^2^ = 3.303, *df* = 1 *P* = 0.069	**3.10 (11.29)** **42.25 (80.44)**	***U = 1601.5*** ***P < 0.001***	**126.28 (69.86)** **92.81 (71.26)**	***U = 1716.0*** ***P = 0.027***

Data are shown as *n*, mean *x̄* (SD) or *ρ* = for the correlation coefficient. Significant associations (*P* < 0.05) are written in bold. Data based on all 133 individuals if not otherwise specified. For normally distributed data, the following parametric tests were used: Student’s t test (*t* value, *df*, *P* value) and Spearman Rho correlation (*ρ* value, *P* value). For non-normally distributed data, the following non-parametric tests were used: Mann–Whitney *U* test (*U* value, *P* value), Kruskal–Wallis test (*H* value, *df*, *P* value), *χ*^2^ test (*χ* value, *df*, *P* value), Fisher’s exact test (exact *χ* value, *P* value). Correction of *P* value for multiple testing using the family-wise error rate (FWE) was performed for families of tests within the same category (marked as *P*_FWE_)

*y-GT *gamma-glutamyl transferase, *ALT *alanine aminotransferase, *AST *aspartate aminotransferase, *MCV *mean cell volume of erythrocytes, *ICD-10 *International Classification of Diseases, 10th edition, *F32 *depressive episode, *F33 *recurrent depressive disorder, *F41 *other anxiety disorders, *F60 *specific personality disorders, χ^2^ χ^2^ value; χ_e_^2^ exact χ value. *P P* value, *U U* value, *df *degrees of freedom, *t*(*df*) *t* value with degrees of freedom, *x̄ *mean, *SD *standard deviation, *H H* value

*Current abstinence: no alcohol consumption reported in 30-day timeline follow back; continuous abstinence: no alcohol consumption since the end of qualified withdrawal treatment

### Six-month follow-up measurements

Higher levels of individual self-reported motivation and self-efficacy are presumed to be associated with better treatment outcomes in patients with AD [10, 22, 28]. Moreover, study designs with a written informed consent process for study participation might be prone to selection bias since populations are not comparable with real-world settings [18]. Our approach to modify the classical procedure of written informed consent in a three-step procedure was approved a priori by the local ethics committee: (1) 5.5 months after the end of QWT we used the addresses available in the hospital database to inform all former inpatients who had received QWT for AD in written form about the study. (2) Trained interviewers then attempted to contact all former inpatients by telephone 6 months (+ maximum 10 days) after the end of their QWT. The protocol specified that 10 attempts to reach a patient could be made at different time points during the day, including weekends. Finally, (3) Successfully contacted patients were asked whether they agreed to participate in the study that they had been informed about (i.e. we obtained oral informed consent). Then, the trained interviewers immediately conducted the semi-structured self-report follow-up interview to assess all outcome variables, as follows: (1) continuous abstinence from the end of QWT until follow-up (i.e. no alcohol use [no single drink]; yes/no), (2) duration (in days) of continuous abstinence (i.e. reported achieved days of continuous abstinence with no single drink after the end of QWT; if patients reported continuous abstinence for the full 6 months, the duration of continuous abstinence was recorded as 180 days), (3) daily alcohol consumption based on 30-day TLFB [37]; mean daily alcohol consumption was calculated in grams: ethanol [g] = volume of beverages × 0.8 g/l × alcohol content [in %]/100%) and current abstinence (no alcohol consumption in 30-day TLFB). Finally, the interviewers assessed participant satisfaction with the inpatient QWT programme using the Munich Patient Satisfaction Scale (MPSS-24) [26] and investigated follow-up treatment after QWT.

### Statistical analysis

Statistical analysis was performed with IBM SPSS25.0 software (IBM, Armonk, NY, USA) with a significance level of *α* = 0.05. The Shapiro–Wilk test was used to assess whether data were normally distributed. Data were not transformed and we used both parametric (Student’s *t*-test, Pearson’s *r*) and non-parametric analyses (Mann–Whitney *U* test, Kruskal–Wallis test, *χ*^2^ test, Fisher’s exact test, Spearman’s *ρ*), depending on the specific distribution properties. Correction for multiple testing using the family-wise error rate (FWE) was performed for families of tests within the same category (e.g. education, living situation). However, due to the exploratory nature of our analysis and our pre-defined aim of identifying candidate variables, no overall *P*-value correction was applied. Finally, baseline variables were used to predict both binary and continuous outcome features using logistic and linear regression analysis, respectively. Since our analyses on the relationship of biomarkers on relapse rates have to be considered exploratory rather than confirmatory, power analyses were not conducted.

## Results

### Participant characteristics

Of the 133 former inpatients included in the follow-up assessment (71.4% men, 28.6% women; mean [SD] age: 47.4 [13.0] years), *n* = 59 participants (44.4%) reported being continuously abstinent since the end of their QWT. The mean (SD) duration of continuous abstinence was 111.44 (72.17) days. A total of *n* = 103 participants (77.4%) reported current abstinence. The mean (SD) daily alcohol consumption among all participants, calculated on the basis of TLFB, was 20.47 (57.40) g. The mean [SD] satisfaction score on the MPSS-24 was 22.40 [4.0] (*n* = 132 for satisfaction score; maximum score = 25). The satisfaction score did not differ between participants who were currently abstinent and those who were currently drinking according to TLFB (22.28 [4.04] vs. 22.83 [3.86]; *U* = 2139.5; *P* = 0.945), nor did it differ between continuously abstinent participants and those who had relapsed (22.51 [3.82] vs. 22.32 [4.16]; *U* = 2139.5; *P* = 0.945). Moreover, there was no significant correlation between the satisfaction score and daily alcohol consumption (*ρ* = 0.127; *P* = 0.148) or days of continuous abstinence (*ρ* = 0.026; *P* = 0.766). Table 1 presents all baseline subject characteristics and outcome results.

### Baseline sociodemographic and clinical variables associated with outcome variables

Several sociodemographic and clinical variables differed significantly between abstinent and relapsed participants or correlated significantly with days of continuous abstinence or current daily alcohol consumption (Table 1). With regard to sociodemographic variables, a higher educational level was associated with both a higher probability for current abstinence (*P* = 0.010) and a lower alcohol consumption (*P* = 0.011). Furthermore, employment status was associated with longer continuous abstinence (*P* = 0.036). With regard to clinical variables, participants with current abstinence had received less oxazepam as withdrawal medication (*P* = 0.041). Higher oxazepam use during withdrawal treatment significantly correlated with higher daily alcohol consumption at the 6-month follow-up (*P* = 0.028). A higher number of previous detoxifications and alcohol treatments correlated with a lower number of days of continuous abstinence (*P* = 0.009). Moreover, the breath alcohol level at admission was lower in participants with current abstinence at the 6-month follow-up (*P* = 0.046). Higher breath alcohol levels at admission correlated with higher mean daily alcohol consumption at the 6-month follow-up (*P* = 0.022).

### Baseline blood biomarkers associated with outcome variables

No single blood biomarker at admission significantly differed between abstinent and relapsed participants or correlated with any outcome variable. We did observe a significant correlation between the AST:ALT ratio and daily alcohol consumption at the 6-month follow-up (*r *= 0.232; *P* = 0.007). A total of *n* = 59 (44.4%) participants were high-risk patients, i.e. they fulfilled the above-mentioned risk threshold of an AST:ALT > 1.00 and MCV > 90.0 fl [17]. At the 6-month follow-up, *n* = 65/74 (87.8%) of the low-risk patients were currently abstinent compared with only *n* = 38/59 (64.4%) of the high-risk patients (*P* = 0.001). Moreover, mean (SD) daily alcohol consumption was significantly higher among high-risk (42.25 [80.44] g) than in low-risk patients (3.10 [11.29], *p* < 0.001). Finally, high-risk patients achieved a shorter period of continuous abstinence (92.81 [71.26] days) than low-risk patients (126.28 [69.86] days, *P* = 0.027) (Table 1).

### Ranking of baseline variables associated with outcome variables

For each baseline variable, we determined whether it was significantly associated with each of the four outcome variables, i.e. continuous abstinence, duration (in days) of continuous abstinence, daily alcohol consumption based on TLFB and current abstinence. Table 2 highlights the ranking of the number of significant associations between the various baseline factors and the four outcome variables. The combination criterion of AST:ALT > 1.00 and MCV > 90.0 fl at baseline was significantly associated with 3 of the 4 outcome variables; the baseline variables educational level, alcohol breath concentration and amount of used withdrawal medication were each significantly associated with 2 of the 4 outcome variables; and the AST:ALT ratio, number of previous inpatient treatments and employment status were significantly associated with 1 of the 4 outcome variables.

**Table 2:** Ranking of baseline variables dependent on the number of significant associations with outcome variables

Baseline factor	No. of associations between baseline factor and outcome variable	Outcome variable			
		Current abstinence*	Continuous abstinence*	Daily alcohol consumption	Days of continuous abstinence
AST:ALT > 1.00 + MCV > 90.0 fl	3	***P = 0.001***	*P* = 0.069	***P < 0.001***	***P = 0.027***
Education level	2	***P = 0.010***	–	***P = 0.011***	–
Breath alcohol level		***P = 0.046***	*P* = 0.088	***P = 0.022***	*P* = 0.068
Dose of oxazepam needed		***P = 0.041***	–	***P = 0.028***	–
AST:ALT ratio	1	*P* = 0.087	–	***P =0 .007***	–
Previous inpatient treatment		–	*P* = 0.058	–	***P = 0.009***
Employment status		–	*P* = 0.091	–	***P = 0.036***
MCV	0**	–	*P* = 0.067	–	*P* = 0.079
Previous alcohol-related seizure		*P* = 0.082	–	*P* = 0.068	–
Psychiatric comorbidity		–	–	–	*P* = 0.066
Type of discharge		–	–	*P* = 0.069	–

Significant *P* values (*P* < 0.05) are written in bold. Non-significant *P* values < 0.1 are specified, but *P* values > 0.1 are indicated as “–”.

*AST *aspartate aminotransferase, *ALT *alanine aminotransferase, *MCV *mean cell volume of erythrocytes

*Current abstinence: no alcohol consumption reported in 30-day timeline follow-back; continuous abstinence: no alcohol consumption since the end of inpatient withdrawal treatment

**Baseline variables with no significant associations and a *P* value ≤ 0.1 are included in the table, but baseline variables with no significant associations and a *P* value >0 .1 are not

### Regression analysis

To investigate effect sizes and to adjust for intervariable confounding effects, we performed regression analysis with those variables that were significantly associated with the respective outcome variable from Table 1. We performed logistic regression to investigate the relationship between the identified baseline variables and current abstinence. Individuals who did not fulfill the combination criterion of AST:ALT > 1.00 and MCV > 90.0 fl at baseline had a 3.52 higher odds ratio to achieve abstinence 6 months after treatment than patients who fulfilled the criterion. Besides the combination criterion, also higher education provided significant higher odds ratios compared to basic school education (Table 3). By performing linear regression analysis for the continuous outcome variables, the fulfilled combination criterion significantly explained parts of the outcome variances of daily alcohol consumption (effect size = 0.347) and days of continuous abstinence (effect size = − 0.190). Besides that, only breath alcohol level at admission significantly predicted daily alcohol consumption 6 months later and number of previous inpatient treatments significantly explained parts of the variance of the achieved days of continuous abstinence (Table 4).

**Table 3:** Logistic regression of the relationship between identified baseline variables on current abstinence

Predictors for current abstinence (logistic regression)	Wald	*df*	Sig	Exp(B)	95% C.I. for EXP(B)	
					Lower	Upper
Breath alcohol level	0.836	1	0.361	0.720	0.355	1.457
Education	11.021	3	**0.012**			
Secondary school to end of year 10^a^	3.901	1	**0.048**	**3.892**	1.010	14.992
Secondary school to end of year 12/13^a^	0.227	1	0.634	0.728	0.196	2.696
University/college^a^	6.362	1	**0.012**	**5.665**	1.472	21.801
AST:ALT > 1.00 + MCV > 90.0 fl^b^	6.154	1	**0.013**	**3.520**	1.302	9.513
Dose of oxazepam needed	0.466	1	0.495	0.998	0.992	1.004

Logistic regression model of the relationship between identified baseline and current abstinence. Independently significant associations (*P* < 0.05) are written in bold. Model statistics: *χ*^2^ = 22.723, *df* = 6, *P* < 0.001, Nagelkerke’s *R* square = 0.262

*Df* degrees of freedom, *Wald χ*^2^ value, Sig. = two-tailed *P* value

^a^Educational level vs. “secondary school to end of year 9”

^b^Not fulfilled vs. fulfilled

**Table 4:** Linear regression between identified baseline variables and daily alcohol consumption and continuous abstinence

	Standardized coefficients beta	t	Sig
(A) Predictors for daily alcohol consumption (linear regression)			
Breath alcohol level	0.192	**2.040**	**0.044**
Education	− 0.160	− 1.894	0.061
AST:ALT ratio	− 0.108	− 1.108	0.270
AST:ALT > 1.00 + MCV > 90.0 fl—fullfilled	0.347	**3.534**	**0.001**
Dose of oxazepam needed	0.138	1.461	0.147
(B) Predictors for days of continuous abstinence (linear regression)			
AST:ALT > 1.00 + MCV > 90.0 fl—fullfilled	− 0.190	− **2.177**	**0.031**
Previous inpatient treatment	− 0.219	− **2.525**	**0.013**
Employment status—Yes	0.048	0.548	0.584

(A) Linear regression model of the relationship between identified baseline variables on daily alcohol consumption. Independently significant associations (P < 0.05) are written in bold. Model statistics: *F* = 6.092, *P* < 0.001, *R* square = 0.209. (B) Linear regression of the relationship between identified baseline variables on days of continuous abstinence. Model statistics: *F* = 4.993, *P* = .003, *R* square = 0.107

## Discussion

The aim of our naturalistic longitudinal study in people who had received extended alcohol withdrawal treatment was to analyse the potential of individual and combined routine blood biomarkers and clinical and sociodemographic variables to predict outcome 6 months later. 48.8% of inpatients were successfully contacted and interviewed 6 months after withdrawal treatment, whereas 51.2% could not be reached. A significant difference between the two groups could only be detected for two baseline parameters (migration status and liver disease detected by ultrasound). All other baseline features including blood biomarkers did not significantly differ between the follow-up group and the non-follow-up group. Hence, by receiving informed consent not during withdrawal treatment but 6 months later during the follow-up survey, we were able to reduce potential selection bias compared to standard a priori written informed consent procedures [18]. Of note, a previous study in an outpatient setting also assessed AST, ALT, GGT and MCV with a 1-year follow-up period in a longitudinal naturalistic approach in patients with AD. This study used written informed consent before study participation and reported a subsequent dropout rate of 44% [14].

A higher educational level and being employed (as sociodemographic baseline variables) and less use of withdrawal medication, fewer previous hospitalizations and lower breath alcohol levels at admission (as clinical baseline variables) were significantly associated with better treatment outcome (Table 1). Even though no single baseline blood biomarker at admission was significantly associated with one outcome variable, the AST:ALT ratio significantly correlated with daily alcohol consumption at the 6-month follow-up. The combined threshold criterion of an AST:ALT > 1.00 and MCV > 90.0 fl was significantly associated with outcome, i.e. significantly more participants who did not fulfil the combination threshold criterion (low-risk patients) achieved current abstinence after 6 months than participants who fulfilled it (high-risk patients). High-risk patients achieved continuous abstinence for a significantly shorter period of time than low-risk patients and had a significantly higher mean daily alcohol consumption (Table 1). Moreover, the combination criterion was significantly associated with more outcome variables (3 out of 4) than any other biological and non-biological factors (Table 2). Finally, we performed a regression analysis to estimate effect sizes or odd ratios and to adjust for confounding within the tested variables. Regression analysis highlighted that the combination criterion significantly predicted the three associated outcome variables. Moreover, all regression models that were based on the significant baseline variables from Table 1 provided significant models to predict part of the respective outcome variance.

In our study, 44.4% of the participants reported being continuously abstinent 6 months after inpatient treatment. This finding is consistent with previous studies reporting a relapse rate of approximately 60% after alcohol treatment [11, 27]. Also in line with previous studies was our finding that the number of previous detoxifications [39] and sociodemographic factors such as employment status and a higher level of education [38] were associated with better treatment outcomes. However, a direct comparison with other studies is not possible, due to several differences between studies (e.g. treatment setting, study design and follow-up period).

In the current study, individual blood biomarkers were not significantly associated with the outcome variables and only the baseline AST:ALT ratio significantly correlated with daily alcohol consumption at the 6-month follow-up. However, we identified the combined threshold criterion of an AST:ALT ratio > 1.00 and MCV > 90.0 fl as a potentially powerful tool to identify patients with a lower and higher risk for relapse after withdrawal treatment. We did not use the threshold criterion from Kawachi et al. [17] as a screening tool to identify patients with AD, but as a potential predictor for future outcomes in patients with AD. It should be noted that at admission all participants met the criteria for AD according to ICD-10, but less than half of them met the threshold cut-offs of Kawachi et al. that were originally applied to identify AD patients. Remarkably, the combined threshold criterion was significantly associated with 3 out of 4 outcome variables and thus outperformed all other baseline variables (Table 2). High-risk patients reported current alcohol consumption significantly more often at the 6-month follow-up, were continuously abstinent for significantly shorter periods and consumed significantly more alcohol per day compared with low-risk patients. Only continuous abstinence as an outcome variable (which was lower in high-risk patients) did not reach the level of significance, which might be due to the low sample size (Table 1). Remarkably, also when we adjusted for potentially confounding effects of other candidate baseline variables by performing regression analysis, the combination threshold criterion of AST:ALT > 1.00 and MCV > 90.0 fl significantly predicted parts of the outcome variables.

To our knowledge, only a few studies have investigated in cohorts of patients with AUD whether baseline values of routine blood biomarkers are associated with AUD-relevant outcome variables. Maenhout et al. [23] suggested that ALT and GGT were predictors for drunk-driving recidivism. Florez and colleagues used all indirect alcohol blood biomarkers (GGT, AST, ALT, AST:ALT ratio and MCV) that were applied in this study and revealed the potential of indirect alcohol markers [14]. However, they had a more general interpretation of good clinical response without detailed distinction of abstinence or daily alcohol consumption. Moreover, there were two assessment time points: one at baseline before treatment and one after 6 months when the active intervention period was completed. Biomarkers at both time points were tested for their potential to predict the outcome after 18 months. For 18 months’ prediction, 6 months biomarkers were more important than baseline parameters [14]. Moreover, GGT was previously identified as a potential predictor and was part of algorithms that applied GGT among other factors to calculate abstinence from drinking [1, 15, 16]. Our analyses confirmed that biological variables, already determined at admission, might help to predict long-term abstinence and the risk of relapse. However, in our study, only the combination of blood biomarkers was significantly associated with 6-months outcome variables.

A multitude of clinical decision limits can be applied to the De Ritis or AST:ALT ratio [7]. An AST:ALT ratio > 1.0 might be an indicator of liver fibrosis/cirrhosis or a resolving alcoholic hepatitis although > 1.0 but < 1.5 could be also a healthy condition in women, whereas an AST:ALT ratio ≥ 2 in adults is associated with worse prognosis of relevant liver damage [7]. The MCV represents a measure for the average size of erythrocytes and is an established biomarker for alcohol abuse and AD [9]. Nevertheless, elevated MCV levels being above the established upper limit of 90 fl might also be affected by other factors, such as e.g. smoking behaviour, poor nutrition and aging [33]. Despite its low sensitivity as a single biological screening marker to detect alcoholism, MCV can enhance sensitivity to detect alcoholism when combined with other alcohol-specific markers such as e.g. GGT [30]. A previous large US population-based study found that these markers identify very heavy alcohol drinking but fail to identify potentially harmful lower levels of alcohol intake [21].

Despite a range of uncertainty, indirect alcohol blood biomarkers and their combinations could reflect the degree of previous alcohol consumption that also reflects the severity of AD [35]. Moreover, it is very likely that the severity of AD is also linked to the risk of relapse after alcohol withdrawal treatment [6]. Therefore, blood biomarkers might help to identify patients with more severe AD and a higher risk of relapse.

This study has several limitations. We did not assess motivation or self-efficacy in participants, although both are associated with treatment outcome [10, 22, 28]. Moreover, the follow-up data are based on self-reports and did not include collateral reports [41] or biomarkers to validate self-reported drinking behaviour. However, previous studies revealed that self-reported alcohol consumption correlates with blood biomarkers [4, 34]. Furthermore, the sample size was relatively small and is based on only one centre with a limited follow-up period although 6 months’ outcome is a good predictor for 5-year abstinence [40]. Therefore, our findings need to be confirmed in a multicentre study with a larger study population. In this study, we did not assess biomarkers like carbohydrate-deficient transferrin (CDT) or direct-ethanol metabolites such as phosphatidylethanol (PEth), ethyl glucuronide (EtG) or ethylsulfate (EtS) [42]. On the other hand, the use of those specific molecules also entails shortcomings that need to be considered. In contrast to AST, ALT, GGT and MCV that are standard parameters in clinical laboratories, these tests are more expensive and only available in specialized centres [3]. Moreover, the direct biomarkers might be more powerful to detect relapse and monitor abstinence but less powerful to monitor clinically relevant chronic cellular impairments in blood samples that could also reflect the somatic severity of AD.

## Conclusion

In summary, this study highlights that, besides sociodemographic and clinical factors, routinely assessed indirect alcohol biomarkers might be useful in the future prediction of long-term outcome in alcohol withdrawal treatment and the identification of patients who have a more severe degree of AD and/or higher risk of relapse. Previously, Neumann and Spies [31] suggested using defined outcome variables to determine the value of single or combined blood biomarkers for future clinical decision-making algorithms with regard to treatment as usual or intensified treatment. Indirect alcohol biomarkers might support future treatment algorithms in daily routine care. Our study might help to pave the way for personalized medicine in AUD treatment with an evidence-based risk stratification and personalized risk prediction that comprises both biological and non-biological factors to identify high-risk patients who might need more intensified treatment and/or relapse prevention.

## References

[1] Aguiar P, Neto D, Lambaz R, Chick J, Ferrinho P (2012). Prognostic factors during outpatient treatment for alcohol dependence: cohort study with 6 months of treatment follow-up. Alcohol Alcohol.

[2] Alatalo P, Koivisto H, Puukka K, Hietala J, Anttila P, Bloigu R, Niemela O (2009). Biomarkers of liver status in heavy drinkers, moderate drinkers and abstainers. Alcohol Alcohol.

[3] Andresen-Streichert H, Muller A, Glahn A, Skopp G, Sterneck M (2018). Alcohol biomarkers in clinical and forensic contexts. Dtsch Arztebl Int.

[4] Babor TF, Steinberg K, Anton R, Del Boca F (2000). Talk is cheap: measuring drinking outcomes in clinical trials. J Stud Alcohol.

[5] Batra A, Muller CA, Mann K, Heinz A (2016). Alcohol dependence and harmful use of alcohol. Dtsch Arztebl Int.

[6] Blaine SK, Sinha R (2017). Alcohol, stress, and glucocorticoids: from risk to dependence and relapse in alcohol use disorders. Neuropharmacology.

[7] Botros M, Sikaris KA (2013). The de ritis ratio: the test of time. Clin Biochem Rev.

[8] Charlet K, Rosenthal A, Lohoff FW, Heinz A, Beck A (2018). Imaging resilience and recovery in alcohol dependence. Addiction.

[9] Conigrave KM, Davies P, Haber P, Whitfield JB (2003). Traditional markers of excessive alcohol use. Addiction.

[10] Dale V, Heather N, Adamson S, Coulton S, Copello A, Godfrey C, Hodgson R, Orford J, Raistrick D, Tober G, Team UR (2017). Predicting drinking outcomes: evidence from the united kingdom alcohol treatment trial (ukatt). Addict Behav.

[11] Dawson DA, Grant BF, Stinson FS, Chou PS, Huang B, Ruan WJ (2005). Recovery from dsm-iv alcohol dependence: United states, 2001–2002. Addiction.

[12] Degenhardt L, Charlson F, Ferrari A, Santomauro D, Erskine H, Mantilla-Herrara A, Whiteford H, Leung J, Naghavi M, Griswold M, Rehm J, Hall W, Sartorius B, Scott J, Vollset SE, Knudsen AK, Haro JM, Patton G, Kopec J, Carvalho Malta D, Topor-Madry R, McGrath J, Haagsma J, Allebeck P, Phillips M, Salomon J, Hay S, Foreman K, Lim S, Mokdad A, Smith M, Gakidou E, Murray C, Vos T (2018). The global burden of disease attributable to alcohol and drug use in 195 countries and territories, 1990–2016: a systematic analysis for the global burden of disease study 2016. Lancet Psychiatr.

[13] Durazzo TC, Gazdzinski S, Yeh PH, Meyerhoff DJ (2008). Combined neuroimaging, neurocognitive and psychiatric factors to predict alcohol consumption following treatment for alcohol dependence. Alcohol Alcohol.

[14] Florez G, Saiz PA, Garcia-Portilla P, De Cos FJ, Dapia S, Alvarez S, Nogueiras L, Bobes J (2015). Predictors of posttreatment drinking outcomes in patients with alcohol dependence. Eur Addict Res.

[15] Gueorguieva R, Wu R, O'Connor PG, Weisner C, Fucito LM, Hoffmann S, Mann K, O'Malley SS (2014). Predictors of abstinence from heavy drinking during treatment in combine and external validation in predict. Alcohol Clin Exp Res.

[16] Hinton DJ, Vazquez MS, Geske JR, Hitschfeld MJ, Ho AMC, Karpyak VM, Biernacka JM, Choi DS (2017). Metabolomics biomarkers to predict acamprosate treatment response in alcohol-dependent subjects. Sci Rep.

[17] Kawachi I, Robinson GM, Stace NH (1990). A combination of raised serum ast: Alt ratio and erythrocyte mean cell volume level detects excessive alcohol consumption. N Z Med J.

[18] Kho ME, Duffett M, Willison DJ, Cook DJ, Brouwers MC (2009). Written informed consent and selection bias in observational studies using medical records: systematic review. BMJ.

[19] Kokkevi A, Hartgers C (1995). Europasi: European adaptation of a multidimensional assessment instrument for drug and alcohol dependence. Eur Addict Res.

[20] Kravos M, Malesic I (2010). Glutamate dehydrogenase as a marker of alcohol dependence. Alcohol Alcohol.

[21] Liangpunsakul S, Qi R, Crabb DW, Witzmann F (2010). Relationship between alcohol drinking and aspartate aminotransferase: alanine aminotransferase (AST:ALT) ratio, mean corpuscular volume (MCV), gamma-glutamyl transpeptidase (GGT), and apolipoprotein A1 and B in the U.S.. Popul J Stud Alcohol Drugs.

[22] Ludwig F, Tadayon-Manssuri E, Strik W, Moggi F (2013). Self-efficacy as a predictor of outcome after residential treatment programs for alcohol dependence: simply ask the patient one question!. Alcohol Clin Exp Res.

[23] Maenhout TM, Poll A, Vermassen T, De Buyzere ML, Delanghe JR, Group RS (2014). Usefulness of indirect alcohol biomarkers for predicting recidivism of drunk-driving among previously convicted drunk-driving offenders: Results from the recidivism of alcohol-impaired driving (road) study. Addiction.

[24] Mann K, Hermann D (2010). Individualised treatment in alcohol-dependent patients. Eur Arch Psychiatry Clin Neurosci.

[25] McDonald H, Borinskya S, Kiryanov N, Gil A, Helander A, Leon DA (2013). Comparative performance of biomarkers of alcohol consumption in a population sample of working-aged men in russia: the izhevsk family study. Addiction.

[26] Möller-Leimkühler AM, Dunkel R, Müller P, Pukies G, de Fazio S, Lehmann E (2002). Is patient satisfaction a unidimensional construct?. Eur Arch Psychiatry Clin Neurosci.

[27] Monahan SC, Finney JW (1996). Explaining abstinence rates following treatment for alcohol abuse: a quantitative synthesis of patient, research design and treatment effects. Addiction.

[28] Moos RH, Moos BS (2006). Rates and predictors of relapse after natural and treated remission from alcohol use disorders. Addiction.

[29] Morley KC, Luquin N, Baillie A, Fraser I, Trent RJ, Dore G, Phung N, Haber PS (2018). Moderation of baclofen response by a gabab receptor polymorphism: Results from the bacald randomized controlled trial. Addiction.

[30] Mundle G, Ackermann K, Munkes J, Steinle D, Mann K (1999). Influence of age, alcohol consumption and abstinence on the sensitivity of carbohydrate-deficient transferrin, gamma-glutamyltransferase and mean corpuscular volume. Alcohol Alcohol.

[31] Neumann T, Spies C (2003). Use of biomarkers for alcohol use disorders in clinical practice. Addiction.

[32] Noone M, Dua J, Markham R (1999). Stress, cognitive factors, and coping resources as predictors of relapse in alcoholics. Addict Behav.

[33] Pavanello S, Snenghi R, Nalesso A, Sartore D, Ferrara SD, Montisci M (2012). Alcohol drinking, mean corpuscular volume of erythrocytes, and alcohol metabolic genotypes in drunk drivers. Alcohol.

[34] Polich JM (1982). The validity of self-reports in alcoholism research. Addict Behav.

[35] Rosoff DB, Charlet K, Jung J, Lee J, Muench C, Luo A, Longley M, Mauro KL, Lohoff FW (2019). Association of high-intensity binge drinking with lipid and liver function enzyme levels. JAMA Netw Open.

[36] Saxena S, Thornicroft G, Knapp M, Whiteford H (2007). Resources for mental health: Scarcity, inequity, and inefficiency. Lancet.

[37] Sobell LC, Sobell MB (1992). Timeline follow-back: a technique for assessing self-reported alcohol consumption. Measuring alcohol consumption: psychosocial and biochemical methods.

[38] Sofin Y, Danker-Hopfe H, Gooren T, Neu P (2017). Predicting inpatient detoxification outcome of alcohol and drug dependent patients: the influence of sociodemographic environment, motivation, impulsivity, and medical comorbidities. J Addict.

[39] Soyka M, Schmidt P (2009). Outpatient alcoholism treatment–24-month outcome and predictors of outcome. Subst Abuse Treat Prevent Policy.

[40] Weisner C, Ray GT, Mertens JR, Satre DD, Moore C (2003). Short-term alcohol and drug treatment outcomes predict long-term outcome. Drug Alcohol Depend.

[41] Whitford JL, Widner SC, Mellick D, Elkins RL (2009). Self-report of drinking compared to objective markers of alcohol consumption. Am J Drug Alcohol Abuse.

[42] Wurst FM, Thon N, Yegles M, Schruck A, Preuss UW, Weinmann W (2015). Ethanol metabolites: their role in the assessment of alcohol intake. Alcohol Clin Exp Res.

